# Young Adulthood Outcomes of Joint Mental Health Trajectories: A Group-Based Trajectory Model Analysis of a 13-Year Longitudinal Cohort Study

**DOI:** 10.1007/s10578-021-01193-8

**Published:** 2021-05-31

**Authors:** Aja Louise Murray, Daniel Nagin, Ingrid Obsuth, Denis Ribeaud, Manuel Eisner

**Affiliations:** 1 Department of Psychology, University of Edinburgh, Edinburgh, UK; 2 Carnegie Mellon University, Pittsburgh, PA USA; 3 Clinical Psychology Department, University of Edinburgh, Edinburgh, UK; 4 Jacobs Center for Productive Youth Development, University of Zurich, Zurich, Switzerland; 5 Institute of Criminology, University of Cambridge, Cambridge, UK

**Keywords:** Comorbidity, Group-based trajectory modelling, ADHD, Internalising problems, Externalising problems

## Abstract

Developmental trajectories of common mental health issues such as ADHD symptoms, internalising problems, and externalising problems can often be usefully summarised in terms of a small number of ‘developmental subtypes’ (e.g., ‘childhood onset’, ‘adolescent onset’) that may differ in their profiles or levels of clinically meaningful variables such as etiological risk factors. However, given the strong tendency for symptoms in these domains to co-occur, it is important to consider not only developmental subtypes in each domain individually, but also the joint developmental subtypes defined by symptoms trajectories in all three domains together (e.g., ‘late onset multimorbid’, ‘pure internalising’, ‘early onset multimorbid’). Previous research has illuminated the joint developmental subtypes of ADHD symptoms, internalising problems, and externalising problems that emerge from normative longitudinal data using methods such as group-based trajectory modelling, as well as predictors of membership in these developmental subtypes. However, information on the long-term outcomes of developmental subtype membership is critical to illuminate the likely nature and intensity of support needs required for individuals whose trajectories fit different developmental subtypes. We, therefore, evaluated the relations between developmental subtypes previously derived using group-based trajectory modelling in the z-proso study (n = 1620 with trajectory data at ages 7, 8, 9, 10, 11, 12, 13, 15) and early adulthood outcomes. Individuals with multimorbid trajectories but not ‘pure’ internalising problem elevations showed higher levels of social exclusion and delinquency at age 20. These associations held irrespective of the specific developmental course of symptoms (e.g., early versus late onset versus remitting). There was also some evidence that intimate partner violence acts as a form of heterotypic continuity for earlier externalising problems. Results underline the need for early intervention to address the pathways that lead to social exclusion and delinquency among young people with multiple co-occurring mental health issues.

Developmental trajectories of mental health symptoms can vary considerably from individual to individual; however, previous research has suggested that it is often both possible and useful to parse this heterogeneity into a small number of trajectory groups [1], or ‘developmental subtypes’. These subtypes do not literally exist but provide an efficient summary of variation in developmental trajectories in terms of categories such as ‘adolescent onset’, ‘childhood limited’, ‘early onset/persistent’ and others that can be compared in terms of their correlates to determine if they provide clinically useful subtypes. Data-driven methods such as group-based trajectory modelling or growth mixture modelling are particularly helpful for identifying optimal developmental subtypes because they emerge from the data rather than being defined on the basis of a priori classifications (e.g., using an age cut-off for symptom onsets to define ‘late onset’ versus ‘early onset’) that may not provide optimal summaries of developmental trajectory variation. Further, in these data driven approaches there is a strong emphasis on visualising and interpreting the (possibly non-linear) shapes of the developmental trajectories defining each subtype, which provides a richer characterisation of the subtypes.

For issues such as anxiety, depression, attention deficit hyperactivity disorder (ADHD), and conduct problems the developmental subtypes identified using data-driven methods are often shown to differ with respect to clinically relevant factors such as early risk markers and sequalae [2–11]. For example, individuals with an earlier onset of ADHD symptoms appear to have higher levels of early peer and conduct problems and lower school readiness and are more likely to be male than individuals with a later onset of symptoms [10]. They also appear to be more severely affected in terms of salient dimensions of psychosocial functioning such as reactive aggression and academic attainment by late adolescence [3, 12]. Taken together, these finding suggest that data-driven developmental subtypes can be clinically useful because they may encode key information about symptom causes and potential outcomes for which intervention/support planning may be beneficial.

Considering the developmental trajectories of a single mental health issue in isolation, however, may provide only a limited understanding of mental health development. Symptoms of different mental health issues have a strong tendency to co-occur, even when categorised as belonging to quite distinct families of disorders, such as internalising problems versus externalising problems [13–18]. Whether or not symptoms occur in isolation or with symptoms of other disorders also appears to have implications for the expression, course and outcomes of disorders, with comorbidity tending to associated with different symptom patterns for the primary disorder relative to non-comorbid cases, as well as in differences in prognosis and treatment response [19, 20]. Thus, evaluating developmental trajectory groups defined jointly by multiple commonly co-occurring mental health symptoms is necessary to establish what are likely to be more clinically informative developmental subtypes.

Only a small number of studies have examined trajectory groups jointly defined by multiple mental health issues [21–24] and even fewer have examined the correlates of trajectory group membership. Examining the correlates of trajectory group membership provides critical information on the clinical meaningfulness of the groups by indicating whether trajectory groups differ in factors such as candidate risk markers, outcomes, and treatment responses. In one of the first studies to do this, Patalay et al., (2017) used a parallel process growth mixture model to examine joint trajectory groups of emotional and behavioral problems between ages 7 and 11 in the large normative Millennium Cohort Study [25]. The model they selected included 5 trajectory groups which they labelled ‘low symptoms’ (57% of the sample), ‘moderate behavioral’ (21%), ‘moderate emotional’ (12.5%), ‘high emotional and moderate behavioral’ (5.5%) and ‘high behavioural and moderate emotional’ (4%). They also tested a large number of candidate predictors but only a handful (infant apprehension, maternal age at birth, and maternal psychological distress) differentiated trajectories that primarily involved elevated behavioural versus emotional symptoms.

Murray et al. [23] noted that previous analyses of joint trajectories had focused exclusively on internalising and externalising problems and argued that ADHD was a particularly important dimension of psychopathology to include alongside these domains for two reasons. First, ADHD symptoms are relatively common in childhood and adolescence, with approximately 3–7% affected at clinically significant levels but many more affected at sub-clinical levels owing to the dimensional nature of symptoms [26–28]. Second, ADHD symptoms commonly co-occur with both internalising problems and externalising problems [13, 28, 29]. ADHD symptoms are typically assumed to act as antecedent to both internalising problems and externalising problems [30, 31]; however, anxiety and depression may also engender attention difficulties, suggesting possible bidirectionality [29, 31, 32].

Murray et al. [23], therefore, used the recently developed group-based multi-trajectory model [33] to explore developmental subtypes defined by joint internalising, externalising, and ADHD trajectories across ages 7–15 in the z-proso study [34]. They fit a series of models with differing numbers of groups and concluded that the optimal model was one with six trajectory groups, which they labelled ‘unaffected’, ‘normative maturing’, ‘internalising’, ‘multimorbid late onset’, ‘multimorbid remitting’, and ‘multimorbid with remitting externalising’. These groups are presented in Fig. 1. Reflecting the normative nature of the sample, most participants belonged to one of two groups that were minimally affected by symptoms, i.e., the ‘unaffected’ or ‘normative maturing’ group. The ‘unaffected’ group (32.5% of the sample) showed consistently low levels of all of ADHD symptoms, externalising problems, and internalising problems. The ‘normative maturing group’ (27.9% of the sample) showed an initial elevation of problems in all three domains but the elevation was slight and symptoms declined to reach low levels by mid-adolescence. Like the ‘unaffected’ group, this group was interpreted as not showing concerning levels of problems because it is reasonably common for childhood to ‘mature out of’ elevations in issue such as conduct problems and hyperactivity [35, 36]. There were then four groups showing some elevation of symptoms in at least one domain at some stage of development. The ‘multimorbid late onset’ group (13.5% of the sample) showed initially low levels of symptoms but an escalating trajectory such that they were showing high levels of symptoms by adolescence. The ‘multimorbid remitting’ (12% of the sample) showed the opposite pattern with initially high levels of symptoms in all three domains but with a decline over development. The ‘multimorbid with remitting externalising problems’ group (3.4% of the sample) showed elevated symptoms in all three domains but with a rapid reduction in externalising problems around the transition to adolescence. ADHD symptoms and internalising problems; however, remained stably high for this group. Finally, a (pure) ‘internalising’ trajectory was identified (10.6% of the sample), characterised by stably high levels of symptoms in the internalising problems domain only. The authors noted that most of the developmental subtypes that emerged from the data were characterised by developmental trajectories that tracked each other closely across the three domains (except in the ‘pure internalising’ trajectory, further underlining the tendency for these three domains to show co-occurrence.

**Fig. 1:**
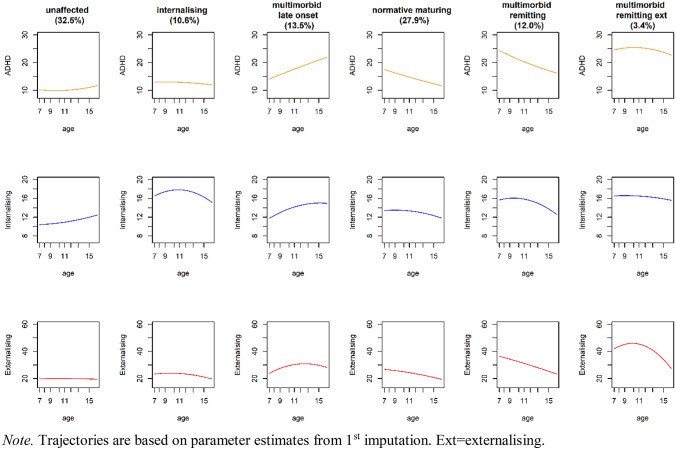
ADHD, internalising and externalising trajectories across ages 7–15

After deriving these groups, Murray et al. [23] compared them with respect to a range of mental health correlates measured either prior to the start of the trajectories (e.g., perinatal factors) or during the trajectories. These analyses suggested that males and victims of bullying were over-represented in trajectory groups characterised by elevated symptoms in multiple domains. Further, maternal post-natal depression was related to groups characterised by symptoms manifesting already in childhood. Low academic achievement and exposure to smoking during pregnancy were related to elevated symptoms irrespective of their particular combination or developmental trajectory. These initial findings suggest that the joint trajectory groups may be differentiable on the basis of established mental health risk markers/correlates. However, this study nor any others have to the best of our knowledge have examined later-life correlates of different developmental trajectories jointly defined by ADHD symptoms, internalising problems, and externalising problems. Understanding the later life correlates associated with developmental trajectories is important in understanding the likely impact, burden, and support needs associated with belonging to particular trajectory groups.

In this study, we aimed to address this gap by examining the age 20 correlates of developmental trajectory subtypes jointly defined by ADHD symptoms, internalising problems and externalising problems at ages 7–15 in the n = 1620 z-proso study sample. There are a wide range of adulthood outcomes that could be (differentially) related to joint mental health trajectory subtypes and it would be impractical to attempt to cover them all. Instead, we focus on a subset of outcomes that have previously been suggested to be related to at least one domain of mental health issues, to contribute to the distress, costs, and impairment associated with these and to be potentially malleable through intervention. We, therefore, examine the links between the previously derived trajectories and social exclusion [37]; subjective stress [38]; violence and criminality [39], and victimisation [40]. These outcomes were measured in early adulthood, 5 years after the endpoint of the estimated developmental trajectories. We hypothesised, based on findings from trajectory analyses of single phenotypes that: (1) membership in a trajectory with any elevation of symptoms in any domain would be associated with poorer adult outcomes than membership in an unaffected group (‘unaffected’ or ‘normative maturing’) (2) membership in a trajectory group characterised by problems in multiple domains (our ‘multimorbid’ groups) would be associated with poorer adult outcomes than membership in less comorbid groups (our ‘internalising’ group). We also explored the differences in early adult outcomes between the remaining groups to provide preliminary insights into possible differences between these groups and that could inform future confirmatory work in independent samples.

## Method

### Ethics

Ethical approval was received from the University of Zurich Faculty of Arts and Humanities ethics committee. The research was conducted in accordance with the ethical standards laid down by the 1964 Declaration of Helskini and its later amendments.

### Participants

Participants were from the Zurich Project on Social Development from Childhood to Adulthood (z-proso) Information on z-proso, including recruitment and assessment procedures can be found in previous publications at: https://www.jacobscenter.uzh.ch/en/research/zproso/aboutus.html. In brief, z-proso is a normative, community-ascertained longitudinal cohort study focused on youth psychosocial development, with data collection beginning in 2004 at age 7 and follow-up waves at ages 8, 9, 10, 11, 12, 13, 15, 17 and 20. The baseline sample was drawn from schools in Zurich, Switzerland and selected based on a random sampling procedure with stratification by school size and location. Participants in 56 schools were invited to take part in the study at the point of entering the first grade.

The majority of children were born between May 1997 and April 1998 at the study baseline. In terms of socioeconomic status, the mean International Socio-Economic Index of Occupational Status (ISEI) score [41] was 44.82, which is approximately equivalent to the occupational prestige level of a book-keeping clerk; SD = 17.75). The sample is diverse in terms of background. A majority of the sample (90%) were born in Switzerland; however, less than half (42.6%) of the participant’s female primary caregivers (and a similar proportion of male primary caregivers) were born in Switzerland. Other than Switzerland, common primary caregiver nations of origin included Germany, Italy, Serbia and Montenegro, Yugoslavia, and Turkey.

From the initial target sample of n = 1675, n = 1620 (52% males) have data relevant for the current study and are, therefore, included in the below-described analyses. Analyses of non-response and attrition have suggested that non-response is largely not related to the substantive variables measured in z-proso, though there is evidence to suggest a slight under-representation of those of an immigrant background [34].

### Measures

#### Mental Health Symptoms

Mental health symptoms of ADHD, internalising, and externalising problems were used in [23] to form the trajectory groups. Symptoms were measured using the *Social Behavior Questionnaire*, which in addition to its general psychometric support [42] has been validated in the present sample [43–45]. For the current study we used the teacher-reported version, which includes eight items measuring ADHD (four measuring inattention and four measuring hyperactivity/impulsivity); seven items measuring internalising problems (four measuring depression and three measuring anxiety); and 15 measuring externalising problems (six measuring oppositional defiant and conduct disorder symptoms and nine measuring aggression). The measure was administered at ages 7, 8, 9, 10, 11, 12, 13, and 15. Responses were recorded on a five-point Likert-type scale from *never* to *very often.* Items were summed to create composite scores of each dimension.

#### Social Exclusion

*Subjective social exclusion* was measured by self-report at age 20 using a six-item scale adapted from [46] measuring (paraphrased): ‘not feeling a part of society’, ‘being segregated’, ‘having no chance in society’, ‘feeling that others depreciate me’, ‘feeling alienated’, ‘feeling worthless for society’. Responses were recorded on a four-point scale from *fully untrue* to *fully true* and summed to form a composite social exclusion score. Omega reliability [47] for this scale was 0.89.

#### Wellbeing

*Subjective stress* was measured by self-report at age 20 using an abbreviated four-item version of the *Perceived Stress Scale* [48, 49]. Items measured ‘felt as if could not control things’, ‘felt nervous/stressed’, ‘felt cannot achieve things’, ‘felt cannot overcome difficulties.’ Responses were recorded on a five-point scale from *never* to *very often*. Item responses were summed to form a composite subjective stress score. Omega reliability for this scale was 0.86.

Wellbeing was additionally measured by self-report at age 20 using a scale labelled *Optimism,* with items: ‘I am very happy and content’, ‘I think life is good’, ‘I am fully of energy’ and ‘I laugh a lot’. Responses were recorded on a four-point scale from *fully untrue* to *fully true*. From these four items a composite was formed by item summation. Omega reliability for this scale was 0.81.

#### Violence and Criminality

*Intimate partner violence (IPV) victimisation* was measured by measured by self-report at age 20 using four subscales capturing physical violence (6 items), sexual violence (4 items), psychological violence (3 items), and monitoring (4 items). The physical violence items were adapted from the Conflict Tactic Scale [50] and measured (paraphrased): ‘slapping/scratching’, ‘biting/kicking’, ‘pushing/grabbing/shoving’, ‘hitting with fist/hand’, ‘threatening with weapon’. The sexual and psychological violence and monitoring scales were adapted from measures previously utilised in two large scale dating violence surveys by [51] and [52]. The sexual violence items measured (paraphrased): ‘pressuring into sex’, ‘touching private parts’, ‘sexual messages’, ‘pressuring to send sexual pictures.’ Psychological violence items measured (paraphrased): ‘insulting in front of others’, ‘putting down regarding looks’, ‘blaming’. The monitoring items measured ‘checking mobile phone’, ‘limiting contacts’, ‘preventing meetings’, and ‘asking about whereabouts.’ Items from each subscale were summed to form composite Physical IPV perpetration, Sexual IPV perpetration, Psychological IPV perpetration, and Monitoring IPV perpetration scores. Items were measured on a four-point scale from *never* to *more than nine times*. Omega reliability scores for the subscales were: 0.71 for physical IPV victimisation; 0.65 for sexual IPV victimisation; 0.67 for psychological IPV victimisation; and 0.85 for monitoring IPV victimisation.

*Intimate partner violence perpetration* was measured by self-report at age 20 with a parallel set of items, worded identically to the perpetration items, except for the fact that they referred to the respondents’ perpetration rather than victimisation experiences. Items from each subscale were summed to form composite Physical IPV victimisation, Sexual IPV perpetration, Psychological IPV victimisation and Monitoring IPV victimisation scores. Omega reliability scores were: 0.76 for physical IPV perpetration; 0.60 for sexual IPV perpetration; and 0.81 for monitoring IPV perpetration.

*Delinquency* was measured by self-report at age 20 using a scale adapted from a previous large-scale survey [53], with additional items developed by the z-proso team. The scale included 24 items measuring (paraphrased): skipping work, stealing at school/university, stealing at work, stealing at home, shoplifting goods worth less than 50 CHF, shoplifting goods worth more than 50 CHF, vehicle theft, driving without a licence, illegal download or upload, car/house burglary, drug dealing, cheating worth more than 100 CHF, driving under the influence of alcohol/drugs, exceeding the speed limit, fare dodging, graffiti, vandalism, carrying a weapon, sexual assault, sexual coercion, threat/extortion, robbery, death threats, and assault. Respondents were asked to indicate whether they perpetrated any of the above-listed on a two-point scale: *yes* versus *no*. Item responses were summed to form a delinquency composite score. Omega reliability for this scale was 0.74.

### Statistical Procedure

#### Imputation Model

We used an imputation model developed in a previous paper to deal with missing data [23]. Multiple imputation is a method for providing a best estimate for what a missing value would have been had it been observed, drawing on available data to inform that estimate. It involves estimating each missing value multiple times to create several imputed datasets, fitting an analysis model in each of the imputed datasets, and then pooling the parameter estimates and their standard errors across the multiple datasets to obtain a single set of model parameter estimates and corresponding standard errors. A primary advantage of multiple imputation is that it takes into account the uncertainty in the parameter estimates due to the necessity of estimating the missing data. This is achieved through incorporating the between-imputation variance in parameters into the pooled estimates of their standard errors.

For the imputation model in the current study (i.e., the model used to create the imputed datasets) all items that would be predictors or outcomes in the main analysis as well as several candidate predictors of trajectories and previously established predictors of participant non-response in the dataset were included [34]. Variables included as candidate predictors of trajectory group membership were maternal smoking during pregnancy, childhood sensation-seeking, maternal post-natal depression, adolescent bullying victimisation, and adolescent academic performance. Variables included as previously established predictors of non-response were: gender, being in a small class at wave 1 (an indicator of special educational needs), neighbourhood social class, primary caregiver language, and teacher-reported prosociality at wave 1. Candidate outcomes of class membership are those outcomes described in the Measures section. Age at each wave was also included in the imputation model because although all participants belonged to the same school year, there was some variation in their age at each wave.

Main analysis variables were imputed at the item level using multiple imputation with chained equations (a fully conditional specification method) in R, implemented using the mice package [54]. The Ns (before imputation) for the variables used in the current study are provided in Table 1. For continuous variables, predictive mean matching was used and for nominal categorical variables a polytomous regression model was used. Items with at least four response options were treated as continuous to reduce computational demands. Three imputations were used and parameter estimates and standard errors were pooled using Rubin’s rules [55]. Three (rather than a large number of) imputations were used because it was necessary to inspect and plot the results from each imputed dataset to check that all three imputations yielded the same groups. Further, due to the involvement of a number of categorical variables we had long estimation times for the imputations. Taken together, this made it impractical to implement a large number of imputations. Nevertheless, multiple imputation would still be preferred over listwise or pairwise deletion methods because it can provide unbiased parameter estimates provided data are missing at random (MAR) [56], that is, conditional on measured scores but not related to unobserved (missing) scores over and above this. Deletion methods; however, rely on the assumption of data being missing completely at random (MCAR). Multiple imputation also provides greater estimation efficiency than methods such as listwise or pairwise deletion even when data are missing completely at random (MCAR) [57].

**Table 1:** Descriptive statistics

	N	Mean	SD	Min	Max	Skew	Kurtosis
ADHD age 7	1312	15.66	7.04	7.00	35.00	0.64	− 0.40
ADHD age 8	1305	14.59	6.91	7.00	35.00	0.81	− 0.16
ADHD age 9	1283	14.34	6.69	7.00	35.00	0.87	0.16
ADHD age 10	1252	14.59	6.96	7.00	35.00	0.84	− 0.11
ADHD age 11	1053	14.31	6.97	7.00	35.00	0.89	0.02
ADHD age 12	970	13.78	6.62	7.00	35.00	1.03	0.41
ADHD age 13	1242	14.08	6.69	7.00	35.00	0.97	0.32
ADHD age 15	1276	13.92	6.56	7.00	35.00	0.91	0.08
Internalising age 7	1302	13.03	5.29	7.00	35.00	0.99	0.72
Internalising age 8	1303	12.43	5.08	7.00	35.00	1.01	0.86
Internalising age 9	1281	12.90	5.19	7.00	35.00	0.85	0.41
Internalising age 10	1240	13.24	5.15	7.00	35.00	0.81	0.30
Internalising age 11	1034	13.31	5.32	7.00	35.00	0.91	0.41
Internalising age 12	967	13.19	5.39	7.00	35.00	0.97	0.74
Internalising age 13	1232	13.13	5.33	7.00	35.00	0.95	0.75
Internalising age 15	1265	13.09	5.27	7.00	35.00	0.92	0.48
Externalising age 7	1263	25.08	9.62	17.00	74.00	1.72	3.01
Externalising age 8	1266	24.67	9.08	17.00	64.00	1.58	2.36
Externalising age 9	1240	25.20	9.45	17.00	68.00	1.68	3.07
Externalising age 10	1213	24.28	9.48	17.00	72.00	1.85	3.64
Externalising age 11	1039	23.75	8.78	17.00	69.00	1.84	3.44
Externalising age 12	953	23.97	9.35	17.00	75.00	1.95	4.18
Externalising age 13	1207	22.28	8.05	17.00	77.00	2.60	8.99
Externalising age 15	1221	22.29	7.79	17.00	85.00	2.38	7.38
Social exclusion age 20	1176	9.12	3.54	6.00	24.00	1.22	1.01
Optimism age 20	1177.00	12.85	2.29	4.00	16.00	− 0.67	0.32
Subjective stress age 20	1180.00	11.28	3.72	4.00	20.00	0.31	− 0.45
Physical IPV perpetration age 20	774.00	6.36	1.03	6.00	19.00	5.24	41.66
Sexual IPV perpetration age 20	774.00	4.04	0.29	4.00	8.00	8.64	86.06
Psychological IPV perpetration age 20	773.00	3.54	0.99	3.00	12.00	2.75	11.56
Monitoring IPV perpetration age 20	774.00	5.93	2.38	4.00	16.00	1.52	2.28
Physical IPV victimisation age 20	774.00	6.57	1.35	6.00	18.00	3.86	20.16
Sexual IPV victimisation age 20	775.00	4.18	0.72	4.00	12.00	6.05	45.20
Psychological IPV victimisation age 20	772.00	3.91	1.47	3.00	12.00	2.15	5.31
Monitoring IPV victimisation age 20	775.00	6.46	2.95	4.00	16.00	1.38	1.37
Delinquency age 20	1174.00	2.47	2.19	0.00	13.00	1.54	3.29

#### Main Analysis

ADHD symptom, internalising problem, and externalising problem scores were used in a group-based multi-trajectory analysis with distal outcomes (GBTM; Nagin, Jones, Passos, & Tremblay, 2018). We adopted the trajectory model used in a previous study described in the introduction [23] and then examined the outcomes of membership of the group identified in this model. The methods underlying the development of this model is described in detail in this previous publication; however, an overview is provided in the section that follows.

#### Multi-trajectory Model

The multi-trajectory model used to summarise the joint developmental trajectories of ADHD symptoms, internalising problems, and externalising problems across ages 7–15 was based on an application of multi-trajectory analysis [23]. Multi-trajectory analysis is a finite mixture modelling method that generalises group-based trajectory modelling to multiple phenotypes [1]. The purpose of group-based trajectory modelling is to identify clusters of individuals in longitudinal data who show similar developmental trajectories on a given phenotype and the purpose of multi-trajectory analysis is then to identify clusters of individuals showing similar trajectories on a set of multiple phenotypes. The trajectories for each phenotype are modelled using polynomial functions of age (or time). To facilitate further analysis and interpretation, an (or small number of) optimal multi-trajectory model(s) must be selected with a given number of trajectory groups. The selection of an optimal model can be informed by model fit statistics, such as information theoretic criteria; however, substantive and practical considerations must also play a role. An example of a substantive consideration would be whether a model with a smaller number of trajectory groups blurred a potentially theoretically important distinction (e.g., lumped remitting and persistent cases together) or whether models with larger numbers of trajectory groups added groups that differed minimally from existing groups. An example of a practical consideration would be avoiding having trajectory groups with very small sample sizes to ensure adequate statistical power for group comparisons in subsequent analysis stages.

A six-class model with both linear and quadratic growth was selected in this previous study as the optimal model. This model was summarised in the Introduction and is presented in Fig. 1. The trajectories were plotted separately for each imputed dataset to ensure that the groups were similar across imputations (pooling to interpret the trajectories would risk masking differences). Figure 1 and the description provided in the Introduction is based on the parameter estimates from the 1st imputation; however, it can be mostly generalised to the 2nd and 3rd imputations as these yielded highly similar groups. The only (minor) difference across imputations was that the ‘internalising’ group showed evidence of elevated ADHD symptoms in the third imputation but had only elevated levels of internalising in the first and second imputed datasets. This may reflect the overlap between anxiety/depression and ADHD in terms of concentration problem symptoms [58].

#### Outcomes of Trajectory Group Membership

Using the multi-trajectory model for ADHD symptoms, internalising problems, and externalising problems developed in [23], mean differences on the outcome variables across trajectory groups were tested by inspecting the between-imputation-variance-adjusted 95% confidence intervals (CIs), i.e., based on pooling by Rubin’s rules [55] for each group mean. Non-overlapping 95% CIs were interpreted as significant group differences. Given the exploratory nature of the study we did not correct for multiple comparisons. The group means and group-based trajectories were estimated in the same model, in a single step. Start values from the estimation of the multi-trajectory model without the outcomes were provided when estimating the models with the outcomes to help ensure that the same trajectory groups emerged in this latter analysis as had been established in the former. This was confirmed by inspection of the model-predicted ADHD symptom, internalising, and externalising problem scores.

## Results

### Descriptive Statistics

Descriptive statistics are provided in Table 1.

### Outcomes of Joint Trajectories

**Fig. 2:**
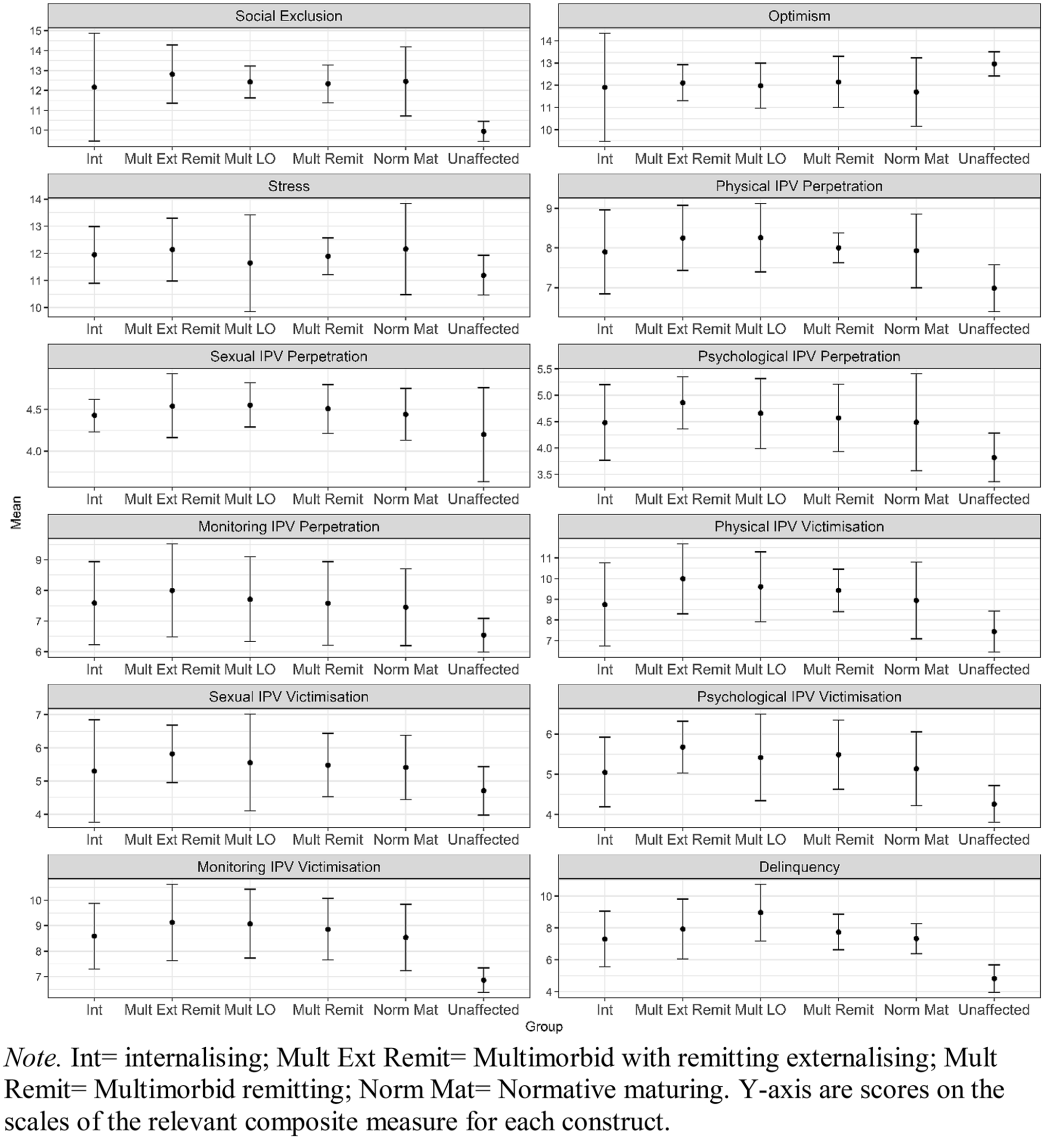
Pooled mean and 95% confidence intervals for outcomes across the six trajectory groups

Group means and 95% CIs for the six trajectory groups on each outcome are provided in Table 2 and visualised in Fig. 2. For social exclusion, the ‘unaffected’ trajectory group differed significantly from all other groups except the internalising group. There were no significant group differences in stress or optimism and only a few differences on IPV. Specifically, the multimorbid remitting group had higher levels of physical IPV perpetration; and the multimorbid with remitting externalising group had higher levels of psychological IPV perpetration than the unaffected group. In addition, the multimorbid with remitting externalising problems group had higher levels of psychological IPV victimisation and the multimorbid late onset, multimorbid remitting and multimorbid with remitting externalising problems groups had higher levels of monitoring IPV victimisation than the unaffected group. Finally, delinquency levels were higher in all groups except the ‘internalising’ group compared to the unaffected group.

**Table 2:** Outcome analyses

Group	Mean	SE	95% CI lower	95% CI upper
*Social exclusion*				
Unaffected	9.94	0.26	9.44	10.45
Internalising	12.16	1.38	9.45	14.87
Multimorbid late onset	12.43	0.41	11.63	13.23
Normative maturing	12.45	0.88	10.72	14.18
Multimorbid remitting	12.33	0.48	11.38	13.27
Multimorbid with remitting externalising	12.81	0.75	11.35	14.28
*Optimism*				
Unaffected	12.96	0.28	12.41	13.51
Internalising	11.91	1.24	9.48	14.34
Multimorbid late onset	11.98	0.52	10.97	12.99
Normative maturing	11.70	0.79	10.16	13.24
Multimorbid remitting	12.15	0.59	11.00	13.30
Multimorbid with remitting externalising	12.11	0.41	11.31	12.92
*Stress*				
Unaffected	11.19	0.37	10.46	11.93
Internalising	11.95	0.53	10.90	12.99
Multimorbid late onset	11.64	0.91	9.85	13.42
Normative maturing	12.16	0.86	10.47	13.84
Multimorbid remitting	11.89	0.35	11.21	12.57
Multimorbid with remitting externalising	12.14	0.59	10.98	13.29
*Physical IPV perpetration*				
Unaffected	6.99	0.30	6.40	7.58
Internalising	7.90	0.54	6.85	8.96
Multimorbid late onset	8.26	0.44	7.40	9.12
Normative maturing	7.93	0.47	7.00	8.85
Multimorbid remitting	8.00	0.19	7.63	8.38
Multimorbid with remitting externalising	8.25	0.42	7.44	9.07
*Sexual IPV perpetration*				
Unaffected	4.20	0.29	3.63	4.76
Internalising	4.43	0.10	4.23	4.62
Multimorbid late onset	4.55	0.13	4.29	4.82
Normative maturing	4.44	0.16	4.13	4.75
Multimorbid remitting	4.51	0.15	4.21	4.80
Multimorbid with remitting externalising	4.54	0.19	4.16	4.93
*Psychological IPV perpetration*				
Unaffected	3.82	0.23	3.36	4.28
Internalising	4.48	0.37	3.77	5.20
Multimorbid late onset	4.66	0.34	3.99	5.32
Normative maturing	4.49	0.47	3.57	5.41
Multimorbid remitting	4.57	0.33	3.93	5.21
Multimorbid with remitting externalising	4.86	0.25	4.36	5.35
*Monitoring IPV perpetration*				
Unaffected	6.54	0.28	5.99	7.09
Internalising	7.59	0.69	6.23	8.94
Multimorbid late onset	7.71	0.71	6.33	9.10
Normative maturing	7.45	0.64	6.20	8.70
Multimorbid remitting	7.58	0.70	6.21	8.94
Multimorbid with remitting externalising	8.00	0.77	6.48	9.52
*Physical IPV victimisation*				
Unaffected	7.44	0.51	6.45	8.43
Internalising	8.75	1.03	6.74	10.77
Multimorbid late onset	9.61	0.87	7.91	11.30
Normative maturing	8.95	0.95	7.09	10.80
Multimorbid remitting	9.44	0.53	8.40	10.47
Multimorbid with remitting externalising	10.00	0.87	8.30	11.69
*Sexual IPV victimisation*				
Unaffected	4.71	0.38	3.97	5.44
Internalising	5.30	0.79	3.76	6.84
Multimorbid late onset	5.56	0.74	4.10	7.02
Normative maturing	5.41	0.49	4.44	6.38
Multimorbid remitting	5.48	0.49	4.53	6.44
Multimorbid with remitting externalising	5.82	0.44	4.96	6.68
*Psychological IPV victimisation*				
Unaffected	4.26	0.23	3.81	4.72
Internalising	5.05	0.44	4.19	5.92
Multimorbid late onset	5.42	0.55	4.34	6.50
Normative maturing	5.14	0.47	4.22	6.06
Multimorbid remitting	5.49	0.44	4.63	6.35
Multimorbid with remitting externalising	5.68	0.33	5.03	6.32
*Monitoring IPV victimisation*				
Unaffected	6.87	0.24	6.39	7.34
Internalising	8.59	0.66	7.30	9.87
Multimorbid late onset	9.07	0.69	7.73	10.42
Normative maturing	8.54	0.66	7.24	9.84
Multimorbid remitting	8.86	0.61	7.66	10.06
Multimorbid with remitting externalising	9.13	0.76	7.63	10.62
*Delinquency*				
Unaffected	4.82	0.44	3.95	5.68
Internalising	7.31	0.89	5.56	9.06
Multimorbid late onset	8.97	0.91	7.18	10.75
Normative maturing	7.33	0.49	6.38	8.28
Multimorbid remitting	7.74	0.57	6.62	8.86
Multimorbid with remitting externalising	7.94	0.96	6.05	9.82

Mean is across imputed datasets; standard errors (SEs) and associated 95% confidence intervals (CIs) are adjusted for between-imputation variation

## Discussion

Mental health issues in different domains, including ADHD symptoms, internalising, and externalising problems tend to co-occur in childhood and adolescence At the same time, it has been illustrated in previous research that the developmental trajectories of ADHD symptoms, internalising, and externalising symptoms can be summarised in terms of a small number of potentially clinically meaningful trajectory groups. However, very few analyses of mental health trajectory groups have taken into account mental health symptom co-occurrence and even fewer have related the trajectory groups identified to potentially clinically meaningful predictors and outcomes. In this study we therefore used group-based trajectory modelling in a large longitudinal sample (n = 1620 measured across 13 years, from childhood to adulthood) to estimate trajectory groups defined by ADHD symptoms, internalising problems and externalising problems and their adulthood outcomes.

We used a model developed in previous research that included six trajectory groups, labelled ‘unaffected’, ‘normative maturing’, ‘internalising’, ‘multimorbid late onset’, ‘multimorbid remitting’, and ‘multimorbid with remitting externalising problems’. Consistent with the community-ascertained nature of the sample, most participants fell into one of the two categories with no or mild and childhood-limited symptoms, i.e., the ‘unaffected’ or ‘normative maturing’ groups. Of the remaining groups, only one (the ‘internalising’ group) had a relatively pure profile in the sense of showing symptom elevations in one domain only. All others showed symptom elevations in all three of internalising, externalising, and ADHD symptoms and usually symptoms were developmentally coupled, i.e., rising and declining in synchrony.

When comparing trajectory groups by age 20 outcomes, there were differences between the ‘unaffected’ group and various combinations of the remaining groups, The unaffected group had lower levels of social exclusion than all groups except the ‘internalising’ group; lower levels of physical IPV perpetration than the ‘multimorbid remitting’ group; lower levels of psychological IPV perpetration than the ‘multimorbid with remitting externalising’; lower levels of monitoring IPV victimisation than the ‘multimorbid late onset’, ‘multimorbid remitting’ and ‘multimorbid with remitting externalising’ groups; lower levels of psychological IPV victimisation than the ‘multimorbid with remitting externalising problems’, and lower levels of delinquency compared to all other groups except the ‘internalising’ groups. Thus, outcomes were generally poorer for individuals with a developmental trajectory characterised by some form of psychopathology elevation, even if that psychopathology was mild and limited to childhood (as in the case of the ‘normative maturing’ group). This supported our hypothesis that elevations of mental health issues would be associated with poorer outcomes in adulthood.

Social exclusion was one of the outcomes most widely associated with developmental trajectories characterised by mental health symptom elevations. This is consistent with previous research that suggests that mental health problems of any type place an individual at increased risk of social exclusion [59]. There are several mechanisms that may link mental health issues in childhood and adolescence to social exclusion. These include reduced motivation and ability to initiate and maintain social relationships due to symptoms and associated features of mental health problems (e.g., anhedonia, low self-esteem); reduced educational attainment, skills acquisition and employment opportunities; increased likelihood of contact with criminal justice system; victimisation; and poverty; and exposure to stigma and discrimination [37, 59, 60]. Further, it is likely that relations between social exclusion and mental health issues are reciprocal, creating a vicious cycle of disadvantage [61]. Of note, social exclusion was not higher in the ‘internalising’ group, consistent with the idea that externalising problems (which were present in all groups with higher social exclusion) are particularly liable to be associated with negative social consequences that may ultimately lead to social exclusion (e.g., McLeod, Uemura, & Rohrman, 2012).

Given the generalised relation between mental health symptom elevations in childhood and adolescence and social exclusion in adulthood, it is important that more research focuses on the mechanisms and potential interventions to prevent social exclusion. These are issues currently addressed by only a small proportion of studies on mental health and social exclusion, which tend to be primarily cross-sectional and/or descriptive in nature [37]. Interventions that reduce stigma, provide additional support in and reduce exclusion from educational settings, combat material disadvantage, build social skills, enhance support networks and increase social participation, and support employment are likely to be promising for reducing social exclusion associated with mental health problems []. Further, given that pathways to disadvantage can begin early in life, early identification of mental health issues and timely access to support is essential for preventing social exclusion in the long term.

Delinquency was also consistently associated with developmental trajectories characterised by mental health symptom elevations, again with the exception of the pure ‘internalising’ profile. Mental health problems, especially externalising problems and ADHD, are more prevalent among young people with delinquency issues as compared to the general population of youth [39, 63, 64]. On the other hand, consistent with its lack of association in the present study, the role of internalising problems in delinquency is less certain, with empirical studies suggesting at best a weak association with youth delinquency [65]. The current study thus provides further evidence that delinquency is a form of heterotypic continuity related to externalising problems and ADHD in childhood and adolescence, with internalising potentially associated with delinquency only due to its tendency to co-occur with ADHD and externalising problems. Our results are also consistent with studies demonstrating strong predictive links between childhood and adolescent ADHD and engagement in criminal behaviour across the lifespan [64, 66], further highlighting the importance of intervening early to prevent escalation to serious delinquency and potential criminal behaviour.

There was some preliminary evidence that intimate partner violence may also be a form of heterotypic continuity, consistent with previous evidence that childhood ADHD and conduct problems are associated with intimate partner violence in adulthood [67–69]. Of note, it was the groups with remitting externalising symptoms that tended to show elevated levels of IPV perpetration, raising the possibility that apparent reductions in aggression merely reflect its redirection towards intimate partners in the transition to adulthood. From a research perspective, this observation underlines the importance of being sensitive to the changing contexts of aggression at different developmental stages when measuring the construct in longitudinal studies [45]. It also highlights the need for continuing support for youth with ADHD and/or externalising problems as they transition to adulthood, as many fundamental interpersonal difficulties are likely to persist, even if their manifestation changes.

Taken together, our results suggest that experiencing multimorbid mental health issues in childhood and adolescence is associated with disadvantage in young adulthood, irrespective of the developmental trajectory of these symptoms. This is consistent with our hypothesis that experiencing multiple issues is associated with poorer outcomes than problems in only one domain; however, the absence of a pure externalising problems or ADHD symptoms group highlights that comorbidity may be much more the norm in relation to these domains. The fact that the young adult disadvantage was observed for trajectories in which symptoms were elevated at different stages suggest that mechanisms that lead to outcomes such as social exclusion and delinquency can operate across multiple stages of child and adolescent development rather than just one critical period.

It is important to consider the limitations of the current study. First, our analyses cannot determine direction of causation and it is thus not clear whether the extent to which the age 20 correlates studied here represent outcomes of developmental trajectories or markers for risk factors. In fact, it is likely that many of the outcomes studied here are reciprocally related to mental health issues. Second, while we did not find strong evidence for unique relations between particular developmental trajectories of mental health issues and specific outcomes, we included only a limited set of outcomes based on available outcomes in the z-proso study. Future studies including a wider range of candidate outcomes may identify unique developmental subtype-to-outcome mappings.

## Conclusions

The developmental trajectories of commonly co-occurring mental health issues in childhood and adolescence can be summarised in terms of a small number of developmental subtypes that differ in dominant symptoms and developmental course. Trajectory groups involving symptom elevations in multiple domains irrespective of the developmental course of symptoms tend to be associated with higher levels of delinquency and social exclusion in early adulthood whereas individuals characterised by problems limited purely to internalising problems are at lower risk.

## Summary

ADHD symptoms, internalising problems, and externalising problems commonly co-occur and also show considerable heterogeneity in terms of their developmental trajectories. In this study we built on previous work that has suggested that individual differences in joint ADHD symptom, internalising, and externalising problems trajectories can be usefully summarised in terms of a small number of ‘developmental subtypes’ and explored whether these subtypes differed in their young adulthood outcomes. Using group-based trajectory modelling of data from the z-proso study (n = 1620 with trajectory data at ages 7, 8, 9, 10, 11, 12, 13, 15), we found that individuals with multimorbid trajectories had higher levels of social exclusion and delinquency at age 20. These associations held irrespective of the specific developmental course of symptoms (e.g., early versus late onset versus remitting). In contrast, individuals with issues characterised by purely elevated symptoms in the internalising problems domain did not show evidence of these outcomes in early adulthood. Results underscore the need for early intervention to address the pathways that lead to social exclusion and delinquency among young people with multiple co-occurring mental health issues.
